# Supplementary material for: Prognostic value of the myocardial salvage index measured by T2-weighted and T1-weighted late gadolinium enhancement magnetic resonance imaging after ST-segment elevation myocardial infarction: A systematic review and meta-regression analysis
Source: PLoS One. 2020 Feb 13;15(2):e0228736. doi: 10.1371/journal.pone.0228736 (PMC7018083; doi:10.1371/journal.pone.0228736)
Supplement: S1 Appendix A — (DOCX) [file pone.0228736.s001.docx]

# **Appendix A. Search terms.**

## **MEDLINE (via PubMed)**

("myocardial infarction" [MESH] OR "myocardial infarction*" OR "myocardial infarct*" OR "MI" OR "cardiac infarction*" OR "cardiac infarct*" OR "heart infarction*" OR "heart infarct*" OR "heart attack*" OR "coronary thrombosis**" OR "deep plaque*" OR "unstable angina*" OR "acute coronary syndrome*" OR "ACS" OR "STEMI" OR “ST elevated” OR “ST elevation” OR “ST segment elevated” OR “ST segment elevation” AND ("magnetic resonance imaging" [MeSH] OR "MRI" OR "magnetic resonance" OR "imaging" OR "MR" OR “CMR”) AND ("T2" OR "T2weighted" OR "T2W" OR "spin spin relaxation") AND ("gadolinium" OR "GD" OR "late enhancement" OR "delayed enhancement" OR "LGE")

## **EMBASE (via Ovid)**

Heart infarction [EMTREE-Thesaurus] OR myocardial infarction$.mp OR myocardial infarct$.mp OR cardiac infarction.mp OR cardiac infarct$.mp OR heart infarction$.mp OR heart infarct$.mp OR heart attack$.mp OR coronary thrombosis$$.mp OR deep plaque$.mp OR unstable angina$.mp OR acute coronary syndrome [EMTREE-Thesaurus] OR acute coronary syndrome$.mp OR ACS.mp OR ST segment elevation myocardial infarction [EMTREE-Thesaurus] OR ST elevated.mp OR ST elevation.mp OR ST segment elevated.mp OR ST segment elevation [EMTREE-Thesaurus] OR STEMI.mp AND nuclear magnetic resonance imaging\[EMTREE-Thesaurus] OR MRI.mp OR magnetic resonance.mp OR imaging.mp AND T2.mp OR T2weighted.mp OR T2w.mp OR spin spin relaxation.mp AND gadolinium\[EMTREE Thesaurus] OR late enhancement.mp OR delayed enhancement.mp OR LGE.mp

## **ISI Web of Science**

Topic=(( "myocardial infarction*" OR "myocardial infarct*" OR "MI" OR "cardiac infarction*" OR "cardiac infarct*" OR "heart infarction*" OR "heart infarct*" OR "heart attack*" OR "coronary thrombosis**" OR "deep plaque*" OR "unstable angina*" OR "acute coronary syndrome*" OR "ACS" OR "STEMI" OR “ST elevated” OR “ST elevation” OR “ST segment elevated” OR “ST segment elevation”) AND ( "MRI" OR "magnetic resonance" OR "imaging" OR "MR" OR “CMR”) AND ("T2" OR "T2weighted" OR "T2W" OR "spin spin relaxation") AND ("gadolinium" OR "GD" OR "late enhancement" OR "delayed enhancement" OR "LGE"
