# Supplementary material for: Prognostic value of the myocardial salvage index measured by T2-weighted and T1-weighted late gadolinium enhancement magnetic resonance imaging after ST-segment elevation myocardial infarction: A systematic review and meta-regression analysis
Source: PLoS One. 2020 Feb 13;15(2):e0228736. doi: 10.1371/journal.pone.0228736 (PMC7018083; doi:10.1371/journal.pone.0228736)
Supplement: S1 Table — (DOCX) [file pone.0228736.s002.docx]

# **Raw data.**

| **Study title** | **First author, Journal, Year** | **Purpose as stated by the study** | **Study design** | **Patient group** | **Patients, n** | **Length of follow-up, months** | **Incidence of MACE during follow-up, % of patients** | **Myocardial salvage index (SD), %** |
| --- | --- | --- | --- | --- | --- | --- | --- | --- |
| Prognosis after ST-elevation myocardial infarction: a study on cardiac magnetic resonance imaging versus clinical routine | de Waha, Trials, 2014 | “This study aimed to evaluate the incremental prognostic value of infarct size, microvascular obstruction, MSI, and LV ejection fraction assessed by cardiac MR imaging in comparison to traditional outcome markers in patients with STEMI reperfused by primary percutaneous intervention.” | Case control study | Occurence of a major cardiovascular event | 52 | 19 | 100 | 25.6 (22.6) |
|  |  |  |  | No occurence of a major cardiovascular event | 226 | 19 | 0 | 66.2 (26.4) |
| Impact of overweigt on myocardial infarct size in patients undergoing primary percutaneous coronary interventions: A magnetic resonance imaging study | Sohn, Atherosclerosis, 2014 | “We evaluated the impact of overweight on myocardial infarct size in patients undergoing primary percutaneous intervention for STEMI.” | Prospective, noninterventional, nonrandomized cohort study | Body mass index beyond 25 | 110 | 6 | 9.9 | 41.1 (26.3) |
|  |  |  |  | Body mass index above 25 | 83 | 6 | 4.8 | 41.8 (20.1) |
| Impact of white blood cell count on myocardial salvage, infarct size, and clinical outcomes in patients undergoing primary percutaneous coronary intervention for ST-segment elevation myocardial infarction: a magnetic resonance imaging study | Chung, Int J Cardiovasc Imaging, 2014 | “We sought to determine the relationship between white blood cell count and infarct size assessed by cardiovascular MR imaging in patients undergoing primary percutaneous coronary intervention for STEMI.” | Prospective, noninterventional, nonrandomized cohort study | Low white blood cell count | 107 | 12 | 0.9 | 46.0 (23.4) |
|  |  |  |  | High white blood cell count | 91 | 12 | 12.1 | 37.5 (21.8) |
| Intracoronary compared with intravenous bolus abciximab application during primary percutaneous coronary intervention in ST-segment elevation myocardial infarction: cardiac magnetic resonance substudy of the AIDA STEMI trial | Eitel, J Am Coll Cardiol, 2013 | “The aim of the AIDA STEMI (Abciximab i.v. Versus i.c. in ST-elevation Myocardial Infarction) cardiac magnetic resonance substudy was to investigate potential benefits of intracoronary versus intravenous abciximab bolus administration on infarct size and reperfusion injury in ST-segment elevation myocardial infarction.” | RCT | Intracoronary abciximab | 394 | 12 | 6.2 | 41.3 (41.3) |
|  |  |  |  | Intravenous abciximab | 401 | 12 | 7.3 | 41.3 (16.4) |
|  |  |  |  | MACE | 53 | 12 | 100 | 38.3 (24.4) |
|  |  |  |  | No MACE | 742 | 12 | 0 | 51.3 (26.7) |
| Right ventricular injury in ST-elevation myocardial infarction: risk stratification by visualization of wall motion, edema, and delayed-enhancement cardiac magnetic resonance | Grothoff, Circ Cardiovasc Imaging, 2012 | “Aims were to determine the predictors and the prognostic significance of right ventricular injury assessed by wall motion abnormalities, edema, myocardial salvage index, and delayed enhancement in acute reperfused STEMI.” | Prospective, noninterventional, nonrandomized cohort study | Right ventricular injury | 69 | 20.9 | 31.9 | 47.0 (20.0) |
|  |  |  |  | No right ventricular injury | 69 | 20.9 | 7.3 | 59.0 (26.0) |
| Distal protection device aggravated microvascular obstruction evaluated by cardiac MR after primary percutaneous intervention for ST-elevation myocardial infarction | Yoon, Int J Cardiol, 2012 | “In a prospective randomized trial, we investigated the mechanism of the poor effect of distal protection and thrombus aspiration in 126 patients with STEMI.” | RCT | Distal protection and thrombus aspiration | 55 | 6 | 1.5 | 31.6 (18.6) |
|  |  |  |  | No distal protection and no thrombus aspiration | 55 | 6 | 3.3 | 27.6 (20.8) |
| A high loading dose of clopidogrel reduces myocardial infarct size in patients undergoing primary percutaneous coronary intervention: a magnetic resonance imaging study | Song, Am Heart J, 2012 | “We sought to determine whether a 600-mg loading dose of clopidogrel reduces myocardial infarct size compared with a 300-mg dose using contrast-enhanced magnetic resonance imaging in patients undergoing primary percutaneous coronary intervention for STEMI.” | Prospective, interventional, nonrandomized cohort study | 300mg Clopidogrel | 81 | 6 | 8.6 | 35.7 (21.1) |
|  |  |  |  | 600mg Clopidogrel | 117 | 6 | 6 | 47.4 (20.4) |
| Cardiovascular magnetic resonance-derived intramyocardial hemorrhage after STEMI: Influence on long-term prognosis, adverse left ventricular remodeling and relationship with microvascular obstruction | Husser, Int J Cardiol, 2013 | “The value of MR imaging-derived intramyocardial hemorrhage for predicting major adverse cardiac events and adverse cardiac remodeling after STEMI and its relationship with microvascular obstruction was analyzed.” | Prospective, noninterventional, nonrandomized cohort study | Occurence of a major cardiovascular event | 47 | 32 | 100 | 22.0 (56.9) |
|  |  |  |  | No occurence of a major cardiovascular event | 257 | 32 | 0 | 27.6 (61.1) |
| Prognostic value and determinants of a hypointense infarct core in T2-weighted cardiac magnetic resonance in acute reperfused ST-elevation-myocardial infarction | Eitel, Circ Cardiovasc Imaging, 2011 | “The aim of this study was to evaluate determinants and prognostic impact of a hypointense infarct core in T2-weighted cardiac MR images, studied in patients after acute, reperfused STEMI.” | Prospective, noninterventional, nonrandomized cohort study | Hypointense core present | 122 | 6 | 16.4 | 32.4 (39.3) |
|  |  |  |  | Hypointense core absent | 224 | 6 | 7 | 59.3 (37.2) |
| Myocardial salvage by CMR correlates with LV remodeling and early ST-segment resolution in acute myocardial infarction | Masci, JACC Cardiovasc Imaging, 2010 | “The purpose of this study was to assess the association of myocardial salvage by cardiac MR imaging with left ventricular LV remodeling and early ST-segment resolution in patients with acute myocardial infarction.” | Prospective, noninterventional case series | All patients | 137 | 4 | 1.5 | 43.8 (48.4) |

MACE: major cardiac events, SD: standard deviation.
