# Supplementary material for: Prognostic value of the myocardial salvage index measured by T2-weighted and T1-weighted late gadolinium enhancement magnetic resonance imaging after ST-segment elevation myocardial infarction: A systematic review and meta-regression analysis
Source: PLoS One. 2020 Feb 13;15(2):e0228736. doi: 10.1371/journal.pone.0228736 (PMC7018083; doi:10.1371/journal.pone.0228736)
Supplement: S2 Table — (DOCX) [file pone.0228736.s003.docx]

# **Risk of bias in individual studies.**

| **Study title** | **First author, Journal, Year** | **Purpose as stated by the study** | **Study design** | **Applied quality assessment tool** | **Result** |
| --- | --- | --- | --- | --- | --- |
| Prognosis after ST-elevation myocardial infarction: a study on cardiac magnetic resonance imaging versus clinical routine | de Waha, Trials, 2014 | “This study aimed to evaluate the incremental prognostic value of infarct size, microvascular obstruction, MSI, and LV ejection fraction assessed by cardiac MR imaging in comparison to traditional outcome markers in patients with STEMI reperfused by primary percutaneous intervention.” | Case control study | Newcastle Ottawa Quality Assessment Scale for Case Control Studies | 8/9 stars  Reason for not reaching the full score: This retrospective analysis is based on patients that were in part initially enrolled in the LIPSIA N-ACC trial and the LIPSIA-STEMI trial. It is unclear whether the dropouts were similarly distributed in the newly created case and control groups for this retrospective study. |
|  |  |  |  |  |  |
| Impact of overweigt on myocardial infarct size in patients undergoing primary percutaneous coronary interventions: A magnetic resonance imaging study | Sohn, Atherosclerosis, 2014 | “We evaluated the impact of overweight on myocardial infarct size in patients undergoing primary percutaneous intervention for STEMI.” | Prospective, noninterventional, nonrandomized cohort study | Newcastle Ottawa Quality Assessment Scale for Cohort Studies | 8/9 stars  Reason for not reaching the full score: A length of follow-up of more than 6 months would in our opinion be more suitable for estimating the risk of MACE. |
|  |  |  |  |  |  |
| Impact of white blood cell count on myocardial salvage, infarct size, and clinical outcomes in patients undergoing primary percutaneous coronary intervention for ST-segment elevation myocardial infarction: a magnetic resonance imaging study | Chung, Int J Cardiovasc Imaging, 2014 | “We sought to determine the relationship between white blood cell count and infarct size assessed by cardiovascular MR imaging in patients undergoing primary percutaneous coronary intervention for STEMI.” | Prospective, noninterventional, nonrandomized cohort study | Newcastle Ottawa Quality Assessment Scale for Cohort Studies | 9/9 stars |
|  |  |  |  |  |  |
| Intracoronary compared with intravenous bolus abciximab application during primary percutaneous coronary intervention in ST-segment elevation myocardial infarction: cardiac magnetic resonance substudy of the AIDA STEMI trial | Eitel, J Am Coll Cardiol, 2013 | “The aim of the AIDA STEMI (Abciximab i.v. Versus i.c. in ST-elevation Myocardial Infarction) cardiac magnetic resonance substudy was to investigate potential benefits of intracoronary versus intravenous abciximab bolus administration on infarct size and reperfusion injury in ST-segment elevation myocardial infarction.” | RCT | Cochrane Risk of Bias Tool | No score.  Random sequence allocation (selection bias):   - Judgement: Low risk of bias - Quote: "randomly assigned in a 1:1 ratio to intracoronary or intravenous abciximab" - Comment: Random allocation was likely performed.   Allocation concealment (selection bias):   - Judgement: Unclear risk of bias - Quote: No quote - Comment: Not described in sufficient detail   Blinding of participants and personnel (performance bias):   - Judgement: Unclear risk of bias - Quote: No quote - Comment: Not described in sufficient detail   Blinding of outcome assessment (detection bias):   - Judgement: Low risk of bias - Quote: "blinded assessment" - Comment: The images were sent to another site for blinded image analysis, which was likely effective.   Incomplete outcome data (attrition bias):   - Unclear risk of bias - Quote: No quote - Comment: Reasons for losses to follow-up were not described.   Selective reporting (reporting bias):   - Low risk of bias - Quote: No quote - Comment: All outcomes measured listed in the methods section were reported in the results section.   Other bias:   - Low risk of bias - Quote: No quote - Comment: No other bias detected |
|  |  |  |  |  |  |
|  |  |  |  |  |  |
|  |  |  |  |  |  |
| Right ventricular injury in ST-elevation myocardial infarction: risk stratification by visualization of wall motion, edema, and delayed-enhancement cardiac magnetic resonance | Grothoff, Circ Cardiovasc Imaging, 2012 | “Aims were to determine the predictors and the prognostic significance of right ventricular injury assessed by wall motion abnormalities, edema, myocardial salvage index, and delayed enhancement in acute reperfused STEMI.” | Prospective, noninterventional, nonrandomized cohort study | Newcastle Ottawa Quality Assessment Scale for Cohort Studies | 9/9 stars |
|  |  |  |  |  |  |
| Distal protection device aggravated microvascular obstruction evaluated by cardiac MR after primary percutaneous intervention for ST-elevation myocardial infarction | Yoon, Int J Cardiol, 2012 | “In a prospective randomized trial, we investigated the mechanism of the poor effect of distal protection and thrombus aspiration in 126 patients with STEMI.” | RCT | Cochrane Risk of Bias Tool | No score.  Random sequence allocation (selection bias):   - Judgement: Low risk of bias - Quote: "Patients were eligible for randomization if PCI was indicated and if the vessel diameter at the infarct lesion was either known or expected to be more than 3.5 mm, without excessive tortuosity or lesion/vessel calcification, with 3 cm or more of distal vessel available to accommodate the device." - Comment: Random allocation was likely performed.   Allocation concealment (selection bias):   - Judgement: Unclear risk of bias - Quote: No quote - Comment: Not described in sufficient detail   Blinding of participants and personnel (performance bias):   - Judgement: Unclear risk of bias - Quote: No quote - Comment: Not described in sufficient detail   Blinding of outcome assessment (detection bias):   - Judgement: Low risk of bias - Quote: "blinded to the clinical data" - Comment: Blinding was likely effective.   Incomplete outcome data (attrition bias):   - High risk of bias - Quote: No quote - Comment: Only one loss to follow-up in the group without distal protection; however, a length of follow-up of more than 6 months would in our opinion be more suitable for estimating the risk of MACE.   Selective reporting (reporting bias):   - Low risk of bias - Quote: No quote - Comment: All outcomes measured listed in the methods section were reported in the results section.   Other bias:   - Low risk of bias - Quote: No quote - Comment: No other bias detected |
|  |  |  |  |  |  |
| A high loading dose of clopidogrel reduces myocardial infarct size in patients undergoing primary percutaneous coronary intervention: a magnetic resonance imaging study | Song, Am Heart J, 2012 | “We sought to determine whether a 600-mg loading dose of clopidogrel reduces myocardial infarct size compared with a 300-mg dose using contrast-enhanced magnetic resonance imaging in patients undergoing primary percutaneous coronary intervention for STEMI.” | Prospective, interventional, nonrandomized cohort study | Newcastle Ottawa Quality Assessment Scale for Cohort Studies | 8/9 stars  Reason for not reaching the full score: A length of follow-up of more than 6 months would in our opinion be more suitable for estimating the risk of MACE. |
|  |  |  |  |  |  |
| Cardiovascular magnetic resonance-derived intramyocardial hemorrhage after STEMI: Influence on long-term prognosis, adverse left ventricular remodeling and relationship with microvascular obstruction | Husser, Int J Cardiol, 2013 | “The value of MR imaging-derived intramyocardial hemorrhage for predicting major adverse cardiac events and adverse cardiac remodeling after STEMI and its relationship with microvascular obstruction was analyzed.” | Prospective, noninterventional, nonrandomized cohort study | Newcastle Ottawa Quality Assessment Scale for Cohort Studies | 9/9 stars |
|  |  |  |  |  |  |
| Prognostic value and determinants of a hypointense infarct core in T2-weighted cardiac magnetic resonance in acute reperfused ST-elevation-myocardial infarction | Eitel, Circ Cardiovasc Imaging, 2011 | “The aim of this study was to evaluate determinants and prognostic impact of a hypointense infarct core in T2-weighted cardiac MR images, studied in patients after acute, reperfused STEMI.” | Prospective, noninterventional, nonrandomized cohort study | Newcastle Ottawa Quality Assessment Scale for Cohort Studies | 8/9 stars  Reason for not reaching the full score: A length of follow-up of more than 6 months would in our opinion be more suitable for estimating the risk of MACE. |
|  |  |  |  |  |  |
| Myocardial salvage by CMR correlates with LV remodeling and early ST-segment resolution in acute myocardial infarction | Masci, JACC Cardiovasc Imaging, 2010 | “The purpose of this study was to assess the association of myocardial salvage by cardiac MR imaging with left ventricular LV remodeling and early ST-segment resolution in patients with acute myocardial infarction.” | Prospective, noninterventional case series | 18-item tool by Moga et al. | 16/18 questions were answered yes or were not applicable.  Reasons for not reaching the full score: The exact length of follow-up was not stated. Competing interests and sources of support were not reported.  Comment: The item whether the intervention of interest was clearly described, the item whether additional interventions were reported, the item whether all relevant outcomes were measured before and after the intervention of interest, and the item whether the adverse events related to the intervention were reported were not applicable since the study is a noninterventional study. |

RCT: randomized controlled trial.
