# Supplementary material for: Prognostic value of the myocardial salvage index measured by T2-weighted and T1-weighted late gadolinium enhancement magnetic resonance imaging after ST-segment elevation myocardial infarction: A systematic review and meta-regression analysis
Source: PLoS One. 2020 Feb 13;15(2):e0228736. doi: 10.1371/journal.pone.0228736 (PMC7018083; doi:10.1371/journal.pone.0228736)
Supplement: S4 Table — (DOCX) [file pone.0228736.s005.docx]

# **Exploration of heterogeneity.**

| **Dependent variable** | |  |  |  |
| --- | --- | --- | --- | --- |
| Incidence of MACE during follow up, % of patients | | | | |
| **Random effects** |  |  |  |  |
| **Factor** | **τ^2^** | **τ** | **Cochran’s Q test for heterogeneity** | |
|  |  |  | **p** |  |
| study | 54.5 | 7.4 | < 0.001 |  |
| **Fixed effects** |  |  |  |  |
| **Factor** | **Estimate** | **p** | **Lower 95 % CI** | **Upper 95 % CI** |
| (Intercept) | 70.76 | < 0.001 | 61.67 | 79.84 |
| Myocardial salvage index, % | -1.51 | < 0.001 | -1.68 | -1.34 |
| Centered length of follow-up, months | 0.25 | 0.417 | -0.35 | 0.85 |
| Centered age, years | 5.89 | <0.001 | 4.89 | 6.89 |
| Centered prevalence of diabetes, % | 0.16 | 0.553 | -0.373 | 0.696 |
| Centered time between STEMI and MRI, days | 1.36 | 0.392 | -1.75 | 4.46 |
| MRI interpretation |  |  |  |  |
| 2 SD above remote myocardium for delineating myocardial edema on T2-weighted MRI and 5 SD above remote myocardium for quantifying of myocardial necrosis on T1-weighted MRI (reference category) | -- | -- | -- | -- |
| Manual contouring | 11.63 | 0.038 | 0.66 | 22.61 |

MACE: major cardiac events, CI: confidence interval, STEMI: ST-segment elevation myocardial infarction, MRI: magnetic resonance imaging, SD: standard deviation.
