# Supplementary material for: Prognostic value of the myocardial salvage index measured by T2-weighted and T1-weighted late gadolinium enhancement magnetic resonance imaging after ST-segment elevation myocardial infarction: A systematic review and meta-regression analysis
Source: PLoS One. 2020 Feb 13;15(2):e0228736. doi: 10.1371/journal.pone.0228736 (PMC7018083; doi:10.1371/journal.pone.0228736)
Supplement: S1 Fig — (DOCX) [file pone.0228736.s006.docx]

# **Funnel plot.**


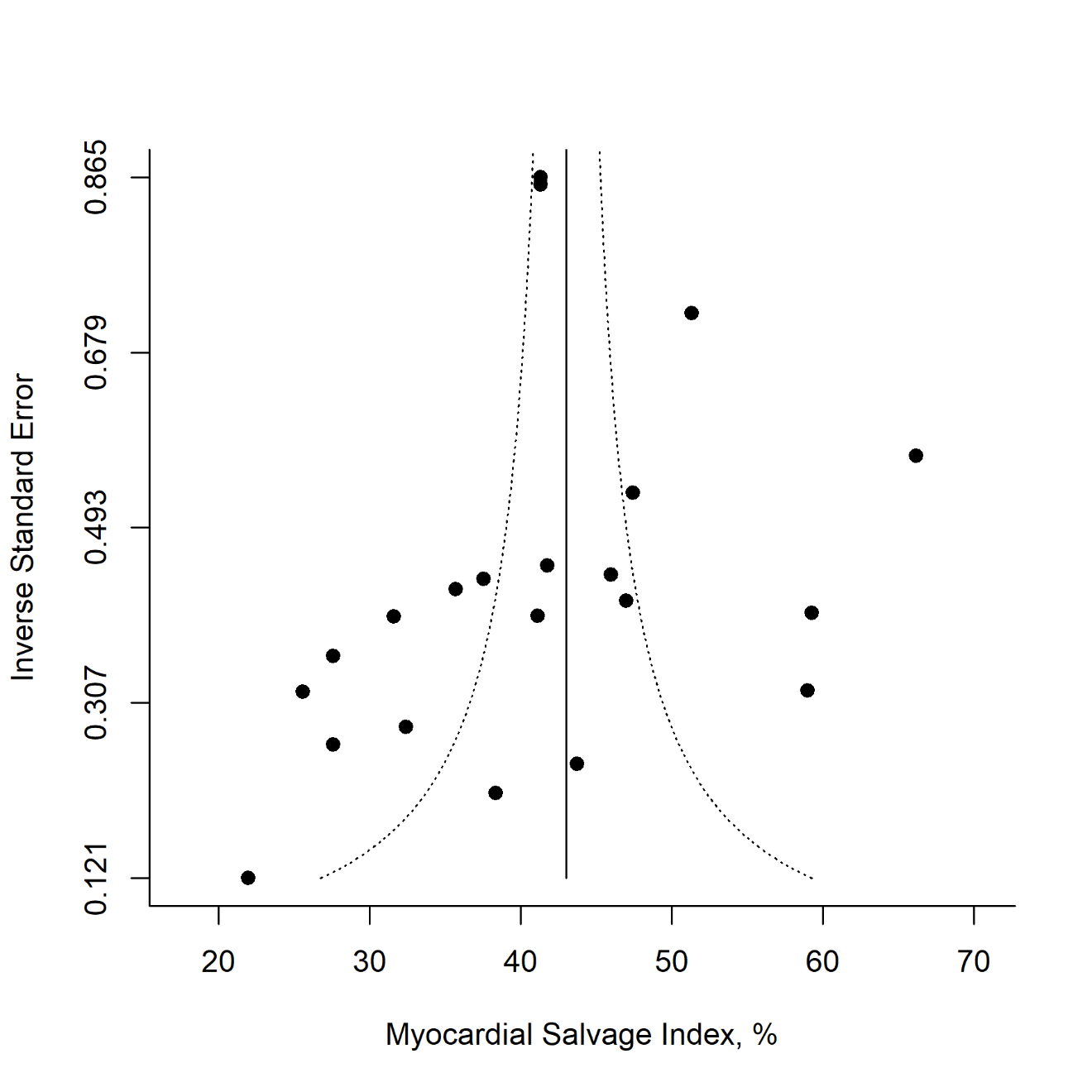


**Fig S1. Funnel plot with the data on the myocardial salvage index measured by T2-weighted and T1-weighted late gadolinium enhancement MRI.** The vertical line represents the pooled value. The dashed lines define the pseudo-95 % confidence region. We did not see apparent asymmetry. MRI: magnetic resonance imaging, CI: confidence interval.
